# Simulation as a toolkit—understanding the perils of blood transfusion in a complex health care environment

**DOI:** 10.1186/s41077-016-0032-z

**Published:** 2016-12-08

**Authors:** Douglas M. Campbell, Laya Poost-Foroosh, Katerina Pavenski, Maya Contreras, Fahad Alam, Jason Lee, Patricia Houston

**Affiliations:** 1 St. Michael’s Hospital, Toronto, ON Canada; 2 University of Toronto, Toronto, ON Canada; 3 Sunnybrook Health Sciences Centre, Toronto, ON Canada; 4 Department of Pediatrics, University of Toronto, St. Michael’s Hospital, 15-014CC, 30 Bond Street, Toronto, ON M5B 1W8 Canada

**Keywords:** Simulation, Medical error, Quality improvement, In situ simulation, Blood transfusion, Patient safety, Latent hazard

## Abstract

**Background:**

Administration of blood is a complex process requiring vigilance and effective teamwork. Despite strict policies and training on blood administration, errors still occur and can lead to mistransfusion with adverse patient outcomes. We used an in situ simulated scenario within an operating room (OR) to identify weaknesses in the current process and hazards that could contribute to mistransfusion.

**Methods:**

A process checklist of critical steps of safe transfusion was developed based on a large academic centre’s internal hospital policy and practice. Ten standardized operating room scenarios were conducted involving management of postoperative bleeding. Scenarios lasted 20 min or until blood transfusion was started. Debriefing followed immediately. Video recordings were reviewed, scored, and evaluated for team performance. Latent safety threats were identified. Focus groups further helped to identify rationale for decisions made. Participants completed questionnaires to evaluate the exercise.

**Results:**

Forty-three experienced OR professionals participated. Of the 19 steps identified as essential for the safe administration of blood components, the median number of steps correctly completed per team was 11. The largest number of errors occurred when different team members interacted and during the immediate pre-transfusion check. We report that this type of learning immediately increased participants’ self-reported ability to perform in a team (90%) and to improve clinical care (88%).

**Conclusions:**

In situ simulation is valuable in identifying common susceptibilities in blood administration error in a complex healthcare organization. Administrators and clinicians may wish to use simulation as an opportunity for system improvement in the delivery of quality care.

## Background

Errors resulting in incompatible transfusions at hospitals have been estimated to occur at a rate of 1 in 38,000–160,000 units transfused [1, 2]. Transfusion errors can have severe consequences for patients including death. A number of factors have been reported to be responsible for transfusion errors including multiple team members involved in distribution of blood components, environmental issues specific to the location of transfusion, overall clinical acuity/distractibility of team members, patient identification issues, and not using a standard checklist [3]. Despite these identified contributors, the majority of transfusion errors are thought to occur immediately prior to point of contact with the patient [4–7]. Other factors which can contribute may depend on local environment or institutional practices [3–6, 8–10]. In order to prevent transfusion errors, most institutions have a pre-transfusion checklist. The compliance of bedside practitioners with this checklist has been shown to be poor and even if done well may not prevent mistransfusion [3].

Communication and teamwork failures underlie the vast majority of adverse events in complex healthcare environments and may be unique to the clinical area itself [11–15]. It is therefore critical to understand the failures within and across teams in addressing errors in particular clinical settings. Interdisciplinary team members may have differing training, experience, knowledge, and value systems [16, 17]. Additionally, members of an interdisciplinary team may not have had the opportunity to train together to manage a crisis situation.

Simulation-based training (SBT) involves immersion of trainees and staff in a “realistic” yet safe environment. SBT has been shown to improve both individual skills and team behavior across a variety clinical disciplines [18–22].

Simulation-based research has also been used to identify safety threats and latent hazards in several clinical environments [23–26]. In situ simulation is increasingly the modality of choice to identify these gaps [27]. It allows teams to practice skills and problem-solve in their actual clinical environments as well as identify contextually specific environmental and/or system threats, also known as latent hazards [26, 27].

Although several studies have incorporated blood transfusion into their simulation scenarios, very few have involved actual interprofessional teams; none have used in situ simulation specifically to improve policy and process for the urgent administration of blood in the operating room (OR) [10, 28–30].

In this exploratory study, we report the use of in situ high fidelity simulation to (1) assess adherence to blood transfusion policy, (2) identify common safety threats when administering blood products in the operating room, and (3) assess the impact of the simulation on participants’ attitudes and beliefs.

## Methods

This study was a collaborative project between the Allan Waters Family Simulation Centre, the Perioperative Services and Transfusion Medicine (TM) Services at St. Michael’s Hospital, Toronto. St. Michael’s Hospital is an academic tertiary care trauma centre located in Toronto, Ontario. The OR environment was chosen as the locale for this study as this was the actual environment in our institution in which a serious transfusion error had been previously reported. The study was reviewed and approved by the institutional Ethics Review Board at St. Michael’s Hospital; informed consent was obtained from all participants.

A simulated scenario involving a postoperative bleed from a recent renal transplant patient was developed and tested. A patient hospital identification (ID) number, hospital electronic record, as well as appropriate blood component labels were generated by the TM laboratory. Bedsides, clinicians used the current hospital Patient Blood Transfusion Record which included a 5-point verification checklist used at the bedside immediately prior to transfusion. The patient hospital card, ID bracelet, hospital chart, and simulated blood components were created by the simulation team. A simulated surgical patient was created using Wireless 3G Sim Man (Laerdal, Norway) with a modified Trauma-Man torso. Changes in vital signs and clinical status could thereby be assessed and acted upon by the OR team in real-time.

Ten interprofessional OR teams including surgical assists, scrub nurses, anesthetists, respiratory therapists, and patient support assistants (PSAs) were invited to participate in the simulation. Each team consisted of one anaesthesiologist and two surgical nurses who worked alongside confederate surgeons (who were familiar with the script and scenario detail). PSAs were also recruited, since in our facility, they are the individuals who retrieve blood components from transfusion medicine. All participants had received training on the new blood transfusion hospital medicine policy by the nurse educator for both perioperative and transfusion medicine departments in the 4 months preceding the study. This included a didactic in-class session, review of the preoperative transfusion policy, and new transfusion medicine checklist. The medical laboratory technologists (MLTs) were aware of the simulated patient and the simulation exercise but were instructed to treat any blood requests as closely as possible to a real situation. All teams were debriefed by the authors (LP, DMC) following the scenarios using a standardized framework with advocacy inquiry technique [30]. All participants were subsequently invited to focus groups to explore the value of the experience and as a surrogate measure of impact.

The scenarios ran until the first simulated red blood component (RBC) was spiked (physically attached to the intravenous system). Simulations were video-recorded including the use of an OR video feed and GoPro, Inc. technology for the PSAs and porters. The time from ordering of blood products to hand-over of blood products to the OR team was recorded.

**Fig. 1:**
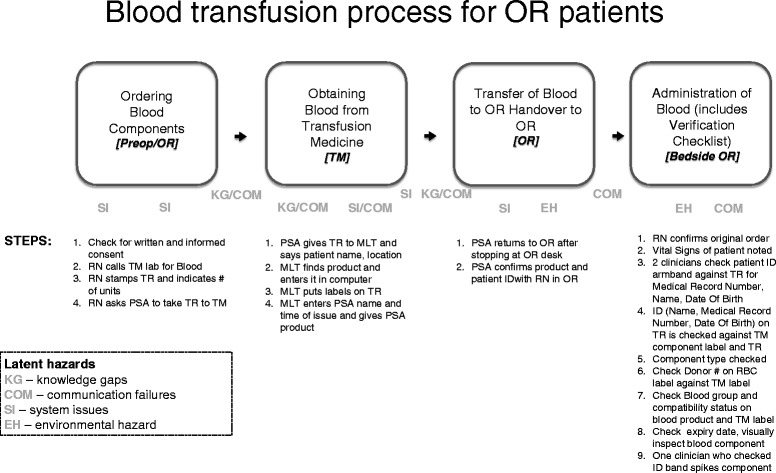
Simulated operating room setup

A team of local experts (Educators, Directors and Managers of TM + Perioperative Services) identified 19 chronological steps required for overall adherence to the hospital policy in the ordering and administration of blood components in the perioperative environment. Adherence to overall blood transfusion policy was scored in a binary fashion (yes or no), with the maximum score for each team to obtain was 19. The overall steps were divided into four categories in order to facilitate error detection: (1) ordering blood components (four steps), (2) obtaining blood components from TM (four steps), (3) transfer of blood components to the OR staff (two steps), and (4) administration of blood components (nine steps) (Fig. 1). The immediate verification checklist used by bedside clinicians was included in this last category (steps 14–18). Events identified as latent hazards were placed into the following categories: knowledge gaps, environmental hazards, communication failures, and system issues as per a previously reported matrix [26]. The videos were reviewed by two members of the research team (DMC,MC) by consensus, in order to identify steps that were completed and/or missed by each team and to classify latent hazards.

### Statistical analysis

Two trained independent raters (PH, FA) reviewed and evaluated performance using two standardized instruments: Anesthesiologist Nontechnical Skills (ANTS) [31] and Clinical Teamwork Scale (CTS) [32]. The ANTS tool is used to address leadership skills within a team, while the CTS represents overall team performance over ten domains, and has been validated in a variety of complex interprofessional health teams. The median and mean scores were calculated for both the ANTS and CTS scales.

Focus groups were conducted 3–6 months following the SBT. A sub-group of participants were probed to discuss their experiences of the SBT, most effective or helpful parts of the SBT, the impact of the SBT on their communication in an interdisciplinary team, and on their attitudes toward teamwork in a crisis situation. Focus groups were audiotaped and transcribed.

Participants subsequently completed the Changes in Inter-professional Attitude Questionnaire (CIAQ) immediately after the simulation and 3 months later to further estimate the impact and retention of the exercise.

The CIAQ questionnaire includes three categories: partnership and shared decision-making, cooperation, and coordination. CIAQ scores were averaged across participants in each of the three categories. The 5-point Likert type responses were averaged to 3-point Likert: (i) agree more than before, (ii) my attitude has not changed, and (iii) disagree more than before. Participants’ responses were summed for each of the four categories. The median and mean scores were calculated for ANTS and CTS scales.

Qualitative data were analyzed using an inductive content analysis [33, 34]. Data were divided into meaning units on the basis of the aim of the study. The meaning units were grouped into codes. Codes with common descriptive content were grouped into categories. Next, the numbers of categories were reduced by combining similar headings into broader categories or themes. Two main categories were generated to provide an overall description of the content of the qualitative data. Research rigor was achieved according to the four criteria of credibility, dependability, conformability, and transferability to ensure trustworthiness [35].

## Results

Ten perioperative teams each composed of one anaesthesiologist (*n* = 9), two nurses (*n* = 17), two confederate surgeons, one technical assistant (TA) (*n* = 4), or patient support assistant (PSA) (*n* = 9) completed the SBT over 2 days. Respiratory therapists (*n* = 2) were also included as requested by teams. The participants had a wide range of experiences in the OR, but the majority had more than 5 years of experience on staff (median 11 years, range 1–44). A subgroup of perioperative participants (five OR nurses, four PSA, two surgical assistants, three anesthesia fellows) in addition to four transfusion medicine staff (one transfusion safety nurse and three medical laboratory technologists) participated in the focus groups.

The median score for correctly following the 19 steps was 11.5 (range 8–13). Only two teams were able to complete 68% of the steps in the blood transfusion (13/19). Eight of 19 steps were typically not performed by the majority of the OR teams, including two steps (steps 17 and 18) which were clearly noted on the Verification Checklist which the teams used in the OR (Table 1). The average time to obtain simulated blood components from the transfusion medicine laboratory was 8.4 min (SD = 1.2 mins).

**Table 1:** Aggregate number of steps followed by all teams

	Step	No. of teams completing steps (max score 10)
Ordering blood components	Check for written and informed consent	2
	RN calls TM lab for blood	9
	RN stamps TR and indicates # of units	6
	RN asks PSA to take TR to TM	10
Obtaining blood from TM	PSA gives TR to MLT and says patient name, location	2
	MLT finds product and enters it in computer	10
	MLT puts labels on TR	10
	MLT enters PSA name and time of issue and gives PSA product	8
Handover of blood to OR	PSA returns to OR after stopping at OR desk	2
	PSA confirms product and ID with RN in OR	3
Administration of blood	RN confirms original order	0
	Vital signs of patient noted	10
	2 clinicians check patient ID armband against TR for medical record number (MRN), name, date of birth (DOB)	4
	ID (Name, MRN, DOB) on TR is checked against TM component label and TR	8
	Component type checked	5
	Check Donor # on RBC label against TM label	10
	Check blood group and compatibility status on RBC and TM label	4
	Check expiry date, visually inspect blood component	3
	One HCP who checked ID band spikes component	10

The largest number of error occurred during handover of the blood components between team members in several environments and when checks were to be repeated (Table 1). There was inconsistent communication between the following teams: PSA and TM staff, PSA and OR nursing staff, and finally OR nurses with anesthetists.

Knowledge and communication gaps were common and included a lack of clarity by PSAs in communicating to TM why blood was ordered and not specifying which OR the patient was in. Other knowledge gaps included poor understanding from all team members regarding why blood components needed to be checked by the OR desk prior to OR arrival. Environmental hazards included OR drapes which were consistently covering the ID band. System issues identified included variant location of the patient consent form for blood transfusion, removal of patient ID card from the OR to stamp requisitions, and delay in retrieval due to elevator wait times (the location of TM lab is on a different floor in our hospital, thereby delaying blood retrieval). Finally, an error relating to communication occurred when the nurse read out loud to the anesthetist; the patient number assigned to the blood product failed to identify a mismatched hospital number in 5 out of 10 scenarios.

Other items of concern discovered during the scenario included retrieval of real un-crossmatched-RBC units from transfusion medicine which were brought into the OR room. In this case, the PSA team insisted on “un-crossmatched” RBC instead of accepting only a “cross-matched” simulated RBC. This highlights the fundamental misunderstanding by the team of when un-crossmatched RBC should be used: un-crossmatched RBCs are to be used only when crossmatched RBCs are not immediately available. Since simulated un-crossmatched RBC was not prepared for this scenario an actual un-crossmatched RBC was released. Secondly, red blood cell units assigned for *different* patients were brought into the same OR on two occasions, which, is against our institutional policy, and, increases the risk of transfusion error. Finally, the patient ID armband was hidden under OR drapes (physical error) and was unused by eight of the ten teams for confirming patient identification. Instead, the team relied on transfusion record, patient chart, etc., which leave and come back to the OR and should not be used for identification pre-transfusion.

The majority of participants perceived partnership, cooperation, and coordination as the strongest elements among their team members during the SBT (Table 2). CAIQ results demonstrated a strong impression that this type of exercise immediately increased participants’ ability to perform in a team, improved clinical care for their patients, and improved role awareness for the participants (Table 3). A chi-square test of independence was calculated comparing AITCS and CAIQ immediately and 3 months post-SBT. There was no significant difference between the immediate and 3 months post-SBT questionnaire results (AITCS: *X* ^2^(1) = 0.99, *p* < .05), (CIAQ: *X* ^2^(1) = 0.98, *p* < .05).

**Table 2:** Assessment of Inter-professional Team Collaboration Scale (AITCS) (*n* = 41)

Sub-scales	Always (%)	Most of the time (%)	Occasionally (%)	Rarely (%)	Never (%)
Partnership and Shared Decision making	27	52	19	2	0
Cooperation	30	51	16	2	1
Coordination	24	46	27	2	1

**Table 3:** Changes in Interprofessional Attitudes Questionnaire (CIAQ) (*n* = 41)

Sub-sategories (Interprofessional training…)	Agree more than before (%)	No change in attitude (%)	Disagree more than before (%)
Promotes team work	87	13	0
Improves clinical and social care	83	16	1
Clarifies/develops team members roles	87	13	0
Not relevant/nothing to learn	15	21	64

The mean overall score for the ANTS was 2.7 (SD = 0.8, range 1.2 to 3.8, max 4). The overall score for CTS was *M* = 5.1 (*SD* = 1.6, range 3 to 7) with 0 = Unacceptable, 1,2,3 = Poor, 4,5,6 = Average, 7,8,9 = Good, and 10 = Perfect. The inter-rater reliability for ANTS and CTS were *r* = 0.39 and *r* = 0.30, respectively, indicating poor inter-rater reliability. Individual raters’ score for ANTS and CTS are summarized and presented in Table 4.

**Table 4:** CTS and ANTS overall and subscale scores (*n* = 10)

Scales	Mean		Median		SD	
	Rater 1	Rater 2	Rater 1	Rater 2	Rater 1	Rater 2
Clinical Teamwork Scale (CTS)						
Communication	5.4	4.4	5.4	4.6	2.0	1.2
Situational awareness	5.3	5.1	5.0	4.5	2.0	1.7
Decision-making	5.4	5.3	5.0	5.0	2.3	1.4
Role responsibility	5.0	3.9	4.3	3.3	1.6	1.2
Overall	5.2	5.0	5.0	5.0	2.9	1.4
Anesthesiologist Nontechnical Skills (ANTS)						
Task management	3.1	2.8	3.0	2.0	0.3	1.0
Team working	3.0	2.4	3.0	2.0	0.8	1.0
Situation awareness	3.2	2.6	3.0	3.0	0.4	1.1
Decision-making	2.7	2.2	2.5	2.0	0.7	1.0

CTS score interpretation: 0 = Unacceptable, 1,2,3 = Poor, 4,5,6 = Average, 7,8,9 = Good, and 10 = Perfect, ANTS score interpretation: 1 = Poor, 2 = Marginal, 3 = Acceptable, 4 = Good

### Focus group results

Participants valued the simulated scenarios as a unique educational experience (Table 5). Participants discussed and compared the SBT relative to a real-life operation. Three categories were identified which characterized SBT as valuable in that it was (1) a controlled activity, (2) a focused learning activity, and (3) allowed conscious learning (Appendix 1).

There was a strong indication that SBT highlights the role of each of the team members in an urgent blood transfusion scenario which may otherwise not be clear. Furthermore, participants’ overall perception of the SBT was that this exercise can be used as an assessment *and* a learning tool (Appendix 2). One participant thought it should be integrated into an orientation package for new staff members.

## Discussion

This study revealed that in situ simulation is valuable in identifying potential causes of error for infrequent but high-risk procedures in a complex healthcare environment. Approximately 40% of the overall process steps required for adherence to policy in the administration of blood components were missed; all of which could potentially contribute to adverse events. Even the printed five-step verification checklist available to teams and required immediately prior to transfusion was performed only 60% of the time, a number which is similar to previously reported studies [36]. Significant high-risk errors occurred, even though the participants had just been trained on a specific updated blood transfusion policy. Furthermore, the majority of the participants rated this scenario as less stressful than an actual case, presumably underestimating the number of errors that could occur in a real clinical situation. Our study highlights the frequency with which steps are missed and are likely underreported by clinical teams in real scenarios. Redundancies in the process steps and the two person-check immediately prior to transfusion likely prevent more mistransfusions from occurring.

We identified that the majority of the errors occurred during handoff of the blood components from one team to another and during the period immediately before administration. The latter observation has been reported previously [3]. The debriefings and focus groups identified *why* steps were missed. For example, one PSA did not stop at the main OR desk for another “check” of the blood components because he understood the request from the surgeon to be “urgent”. This “missed step” could have prevented another patient’s blood components from being brought into the same OR. Integration of SBT may therefore be useful in targeting problematic areas for particular teams in understanding and ultimately adhering to important policy steps. Even in our simulated scenarios, unordered components and components destined for other patients were brought into the OR despite an existing policy that prohibited this practice. We used this simulation exercise as an educational opportunity to reemphasize to staff why this is risky practice and to introduce tools to prevent this in the future.

Our study is the first report to use an interprofessional simulated crisis scenario to examine the entire process of obtaining and administering blood to a patient in the OR. Other studies using simulation in blood transfusion were limited in their focus to only one or two disciplines [36–39], involved obstetrical teams investigated a single confounding factor to transfusion error such as distraction [30], or looked only at the usability of components [40].

In our in situ simulated scenarios, the participants valued the educational experience (Table 3). Our teams reported that simulation-based OR team training positively could improve teamwork, increase awareness of colleagues’ roles, and could be a valuable training exercise in critical care environments. This impression persisted even 3 months after the simulation exercise potentially that this educational exercise had a lasting impact.

Interestingly, team performance and team communication as assessed by two independent raters were marked as poor. This is in direct contrast to the self-reported scales of performance by participants. The discrepancy between observed measures of leadership and teamwork and self-assessment has been reported previously in the simulation literature [19]. Interestingly, both video reviewers identified similar deficits in role responsibility (CTS scale) and decision-making (ANTS) (Table 4). Previous reports of interruption and distraction were not specifically examined in our simulations and could further contribute to mistransfussion events [36].

Focus group data suggest that SBT is a controlled, focused, and conscious learning activity that can be used as a combined educational and quality improvement tool. Participants perceived that being open to scrutiny and the potential for embarrassment during the exercise stimulated learning. Finally, our SBT helped participants recognize and appreciate each interdisciplinary team members’ roles and responsibilities in a crisis situation.

It is possible that we underestimated the hazards which could occur in real life, since our study was carried out 2 months following implementation of a new blood transfusion policy. Many of the participants noted that the perceived stress level of the simulated environment was relatively low and that more distractions can occur in a real life operating room which interferes with safe blood transfusion practice.

This study has several limitations. First, the hazards for unsafe blood transfusion practice identified in our “simulated OR” may not translate directly to an actual OR but clearly points to opportunities for targeted education and reflection. We could not assess the performance of all the perioperative team members in our institution, so it is possible that the other teams may have performed better. However, the consistency of the errors from the teams we observed would suggest otherwise. Furthermore, we were unable to have real surgeons participate in the exercise, and their presence may have affected team performance and procedural vigilance. The 19-point Process Checklist we used was specific to our institution and our preoperative team; other institutions may have a different process for obtaining and administering blood products.

Team performance was rated as poor using two different team assessment tools (ANTS and CTS). However, evidence shows that when teamwork and communication skills are learned and practiced, they can lead to improvements in clinical care [41]. Identification of risk and latent hazards in a policy or process provides an opportunity for performance assessment and in our opinion “targeted” education.

Similar studies could also be conducted in other high stakes environment such as intensive care unit or the emergency room, since these environments may have unique stressors and hazards. SBT could be used to assess performance following implementation of new technologies aimed at reducing mistransfusion such as electronic positive patient identification by barcoding. Evidence shows that new computer-based technology is available and may mitigate blood transfusion errors [42]. Arriaga et al. reported the use of novel surgical-crisis checklists in order to improve adherence to gold-standard lifesaving processes [22]. These were mostly clinical algorithms related to unexpected patient deteriorations. It is unclear at this time if readily available checklists that supplement the verification checklists or other cognitive aids could improve adherence to blood transfusion policy in the OR environment, but warrants further study.

Whether the frequency or severity of blood transfusion error decrease over time as a result of the training remains to be seen and may in fact be difficult to assess. However, as a direct result of this initiative, we will be introducing several educational and operational initiatives to minimize the risk of blood component transfusion error. Firstly, other safety initiatives in the hospital (e.g., ER trauma training) will incorporate blood transfusion error analysis. Secondly, scripted communicated guides will be developed and implemented for PSAs to facilitate clear communication among transfusion medicine members and OR staff. Finally, patient ID bands will now be placed on more accessible areas of the patient to facilitate ID band verification prior to spiking of blood components. Our organization is also now considering purchasing frequency devices to ensure safety.

## Conclusions

In summary, in situ simulation is a useful tool in understanding adherence to policy, latent safety hazards in complex hospital environments such as the OR. Clinical staff may not be adhering to existing failures between team members outside the immediate circle of care of the patient, environmental hazards in the OR, knowledge gaps around existing policy recommendations, and system issues unique to hospital layout. Organizations may be particularly interested in in situ simulation as a strategy to identify and amend gaps in existing policy and engage front-line workers in patient safety initiatives.

## Appendix 1: Focus group data

Comparison of SBT to an actual operating room event

### Category 1: Controlled activity

Participants described SBT as a safe environment with no harmful consequences for patients; thus, the activity is less stressful than a real life occurrence; however, there is a different type of stress associated with participating in SBT.

“The PSA that was assigned to my team was like right there and in real life that would not happen… I would have to call for that PSA, even in a crisis situation; they wouldn't be kind of hovering in the background ready to go.”

### Category 2: Focused learning

Participants described SBT as a learning experience with specific learning objective which distinguishes the activity from a real-life occurrence. Team members have fewer tasks relative to a similar real life scenario; as a result, there are fewer distractions which enable participants to focus on the learning objectives of the SBT.

“when you focus on one tiny aspect of your role, and in this aspect it was the delivery of the blood to the operating room, when you're just focused on that one thing and you find out that everyone has a different way of doing things,… so when you have one little tiny aspect and you look at how people deliver their care, it sort of again you know, hyper focuses on situation and how each person interprets their role and their job in that situation and so it highlights maybe problems or ways that we're doing that potentially could be problems.”

### Category 3: Conscious learning

Participants described SBT as a conscious learning experience in terms of what they do and how they do it. Participants compared the SBT setting to a movie set where actors are being watched. One participant described being more alert because he anticipated that there would be an unexpected event in the scenario.

“I thought initially communication was fairly adequate. Actually, it might have been because it was under the Ambit of a witnessed exercise.”

### Participants’ views of the SBT

Participants described SBT as both an assessment and a learning tool. Each of the categories with relevant interview excerpts is described below.

### Category 1: SBT as an assessment tool

Participants described their experience of SBT as positive in terms of realizing potential gaps in a complex interdisciplinary scenario that may lead to an adverse event.

“I felt that the team was very focussed on the scenario and what they were doing and communicated well. I felt in a different OR setting [real life] there would have been more bodies with more distractions and that takes new communication…it [SBT] clarified how well the team can work without the distractors and how distractors can be the thing that really takes form teamwork.”

SBT was also highlighted as a tool for self-assessment and reflection. The distinctive features of SBT enable participants to evaluate their performance in a simulated crisis situation.

“It allows you to step back and assess your performance without any negative connotations or impact for the patient. So I definitely didn’t feel a high level of stress throughout the simulation because the most negative thing that could happen is I could be embarrassed about it and that is really not a big deal in the context of things.”

“it allowed me the time to think throughout of what I was doing or not doing without unnecessarily worrying about the patient so it allowed, by taking the patient out of the dynamic, it allowed me more time to look at the dynamic within the room and realize how I respond to people in that setting and what I could do better and what I don’t do.”

### Category 2: SBT as a learning tool

Participants underlined that SBT is a valuable tool for learning by doing without any actual harm to real patients. SBT was perceived as a tool that facilitates learning of a complex process such as urgent blood transfusion in an interprofessional environment. The training is especially valuable to novice healthcare professionals and those who are new to the organization with limited knowledge of policy and procedures. One participant described how the exercise is a learning experience in task management:

“The most effective parts were actually probably highlighting the steps that actually happened for the blood transfusion. Small things like checking the band like the nurses always do and I probably don’t pay…wasn’t even aware that that was how important it was in that step so I became more aware of the steps within the protocol of what you need to do and that was probably the main thing I got out of it.”

SBT provides an opportunity for the comparison of a complex process between a real-life occurrence and simulated scenario and detecting potential barriers in the real life situation. For example, one participant described her struggle in a real-life situation based on her experience in SBT:

“I think the struggle in that scenario in real life is actually of the anesthetist, not only minding the patient and trying to figure out what is going on, but ensuring that the nurse in the room isn’t being…who has a very important job isn’t being dragged off to do less important jobs by people who don’t realize the priority. In the scenario it was managed quite well but I think in real life, I think that is what happens and that is difficult because you are usually trying to counteract someone more senior who wants to use your key resources.”

In addition to assessment and learning tool, SBT underlines teamwork. Participants perceived SBT extremely valuable in terms of understanding and appreciating each team member’s role in a complex process and realizing how each team member contributes to the process of care. This finding is in line with AITCS and CIAQ results.

“I think it’s very good for understanding the roles of different people in the OR because I’ll be completely honest, I didn’t know what a PSA was…. I found that out from the exercise what actual role was and they are actually a very helpful an important part of the team.”

“I wanted to actually to ask that maybe the scenario or the simulation had you to realize that -- that for example, this scrub nurse might need -- might need a little bit of help. You know, the realization that she needs to have some support.”

## Appendix 2: Focus group summary

**Table 5:** Focus group summary

SBT characteristics	Participants perceptions of SBT	
	Assessment tool	Learning tool
Controlled activity “The PSA that was assigned to my team was right there and in real life that would not happen.”	Detecting gaps and assessing teamwork “it [SBT] clarified how well the team can work without the distractors and how distractors can be the thing that really takes form teamwork.”	Learning by doing without harm to patients
Focused learning activity “I think the communication was great and part of the reason for that was it was hyper focused on the one thing that we were doing and in real life, that doesn't always play out that way.”		Learning a complex process in an interprofessional environment “Within the team I learned how to have a better understanding of how the process of getting the blood into the OR and how it effects where the communication breakdown can occur.”
Conscious learning “The learning was more because I knew I was being evaluated.”	Self-assessment and reflection “It allows you to step back and assess your performance without any negative connotations or impact for the patient.”	

## Abbreviations

ANTS: Anesthesiologists Nontechnical Skills
CIAQ: Changes in Inter-professional Attitude Questionnaire
CTS: Clinical Teamwork Scale
ID: Patient hospital identification number
MLTs: Medical laboratory technologists
OR: Operating room
PSAs: Patient support assistants
RBC: Red blood component
SBT: Simulation-based training
TA: Technical assistant
TM: Transfusion medicine
TR: Transfusion record
